# Complex Role of Hypnotizability in the Cognitive Control of Pain

**DOI:** 10.3389/fpsyg.2018.02272

**Published:** 2018-11-20

**Authors:** Enrica L. Santarcangelo, Sybille Consoli

**Affiliations:** Department of Translational Research and New Technologies in Medicine and Surgery University of Pisa, Pisa, Italy

**Keywords:** hypnotizability, analgesia, BISBAS, cerebellum, expectation, insula, suggestions, pain

This opinion article deals with the role of hypnotizability in the efficacy of the suggestions of analgesia for the cognitive control of pain and of its physiological correlates.

Hypnotizability is a multimensional trait including suggestibility (Raz, 2007) and other characteristics such as fantasy proneness, attitude to be deeply absorped in specific tasks/mental images (Green and Lynn, 2011; Dasse et al., 2015), strong functional equivalence between imagery and perception (Papalia et al., 2014; Santarcangelo, 2014; Ibanez-Marcelo et al., 2018). It predicts the proneness to accept suggestions (Green et al., 2005; Elkins et al., 2015) and is measured by scales so that the general population is classified as high (*highs*, about 15%), medium (*mediums*, about 70%) and low (*lows*, about 15%) susceptible to hypnosis (De Pascalis et al., 2000). In the ordinary state of consciousness and in the absence of specific suggestions, different levels of hypnotizability are associated with different cerebral (Landry et al., 2017), cerebellar (Bocci et al., 2017; Picerni et al., 2018), sensorimotor and cardiovascular characteristics (Santarcangelo and Scattina, 2016).

The paper describes the interaction between explicit suggestions of analgesia and the expectation of pain relief (Huber et al., 2013; De Pascalis and Scacchia, 2016) on the basis of new findings regarding the hypnotisability-related polymorphism of opioid receptors μ1 (Presciuttini et al., 2018). In addition, the paper reports the observed joined influence of hypnotisability and cognitive-emotional traits (Madeo et al., 2015; Jensen et al., 2016) conceptualized as Behavioral Inhibition/Activation System (BIS/BAS) (Gray, 1990) and as Interoception/Interoceptive Awareness (Sebastiani et al., 2018; Varanini et al., 2018) on pain. Finally, the paper proposes that the observed morpho-functional peculiarities of the *highs*' salience network—insula, cingulate and prefrontal cortex (Landry et al., 2017)—and cerebellum (Bocci et al., 2017; Picerni et al., 2018) may play a role in the complex role of hypnotizability in pain modulation. In this respect, it should be noted that, although hypnotisability is an approximately stable individual trait, socio-cognitive factors such as relational disposition and the manipulation of expectation can modulate the efficacy of suggestions (Kirsch, 2018). The possible biological substrates for some of these factors, for instance the oxytocn release during hypnotic interventions, are now emerging (Kasos et al., 2018).

## Hypnotizability and suggestions for analgesia

Suggestions are explicit instructions aimed at modifying perception, memory and behavior (Braffman and Kirsch, 1999). The suggestions for analgesia are widely employed owing to their efficacy in the cognitive control of pain, which can be predicted by the subjects' hypnotizability scores (Dillworth et al., 2012; Enea et al., 2014; Koban et al., 2017).

Hypnotisability scores predict the efficacy of the suggestions for analgesia in both the ordinary state of consciousness—that is in the absence of any procedure of hypnotic induction—and under hypnosis (Milling et al., 2005; Derbyshire et al., 2009; Meyer and Lynn, 2011). Explicit suggestions for analgesia can be associated or not with instructions for relaxation and pleasant imagery (Zachariae and Bjerring, 1994; Carlson et al., 2017; Hamlin and Robertson, 2017). Personalized suggestions (Berna et al., 2012; Koban et al., 2017) can be prepared for each patient according to her/his preference and directed to the sensory (Hofbauer et al., 2001) or cognitive-affective dimension of pain (Rainville et al., 1997) or both (Feldman, 2009). Neuroimaging studies (Del Casale et al., 2015) have shown that in *highs* the suggestions for analgesia modulate the functional connectivity among the regions of the pain matrix being able to modify pain perception, attention to pain, defensive responses and any other component of pain experience and behavior (Faymonville et al., 2003; Zeev-Wolf et al., 2016). EEG and EMG studies have also shown that in *highs* the suggestions for analgesia decrease pain and both cortical activity (De Pascalis et al., 1999, 2015; Valentini et al., 2013) and nociceptive reflexes (Kiernan et al., 1995; Danziger et al., 1998). Also *mediums* may respond to suggestions for analgesia, although to a lower extent (Fidanza et al., 2017). This enhances the number of subjects who may benefit from suggestion-induced analgesia from 15 to 85% of the general population (Montgomery et al., 2002a,b; Milling et al., 2006, 2007).

## Hypnotizability and expectation of pain relief

*Highs* are more responsive than *lows* and *mediums* not only to the explicit suggestions of analgesia, but also to the conditioned analgesia, or Diffuse Noxious Inhibitory Control (Sandrini et al., 2000; Fidanza et al., 2017) which is mediated by endogenous opioids (Granot et al., 2008). This suggests that expectation-induced mechanisms, which are more effective in *highs* than in *lows*, are associated with those sustaining the conditioned analgesia. However, in *highs* the expectation of pain relief does not totally account for the suggestion induced analgesia (Gearan and Kirsch, 1993) and it is unlikely that the expectation of analgesia could be sustained by opioid mechanisms, in contrast to the general population (Amanzio and Benedetti, 1999; Benedetti et al., 1999; Petrovic et al., 2002; Zubieta et al., 2005; Scott et al., 2008; Babel et al., 2017). In fact, not only the effects of suggestions is not abolished by naloxone (Moret et al., 1991) but, in addition, *highs* display the μ1 polymorphism (Presciuttini et al., 2018) which has been found associated with low sensitivity to opiates, low placebo response (Trescot and Faynboym, 2014; Bartošová et al., 2015; Peciña and Zubieta, 2015) and larger opiates consumption for post-surgery (Zhang et al., 2005; Boswell et al., 2013; Sia et al., 2013; Ren et al., 2015) and cancer pain (Gong et al., 2013; Wan et al., 2015; Yao et al., 2015).

In the general population, pain is associated with modulation of the activation and fuctional connectivity of the “pain matrix” that is the brain region sustaining the varions dimensions of pain (Legrain et al., 2011). It includes the primary and secondary somatosensory areas, the insula and the anterior cingulate cortex. The emergence of pain depends on the flow and integration of information among these areas and is a function of indidual characteristics and of the context (Iannetti and Mouraux, 2010).

Both explicit suggestions and placebo responses are due to top-down mechanisms (Zunhammer et al., 2018), but it has been shown that, in correspondence of similar subjective response to expectation-induced placebo, *highs* and *lows* exhibit opposite patterns of activity and functional connectivity (Huber et al., 2013). In fact, the former exhibit reduced functional connectivity between the right dorsolateral prefrontal cortex (rDLPC) and the anterior midcingulate/medial prefrontal cortex, the left inferior frontal gyrus and the right cerebellum. In addition, placebo analgesia is associated with deactivation in the thalamus, basal ganglia, left precuneus and bilateral temporal gyrus only in *highs*. The observed differences are in line with earlier findings indicating that in the general population placebo analgesia is sustained by circuits involved in the regulation of emotional processes (Amanzio et al., 2011).

An observation relevant to clinical interventions, however, is that an experimental session including relaxation or distraction and suggestions for analgesia modulates pain experience also in chronic pain patients with low hypnotizability scores (Carli et al., 2008). This does not challenge the predictive role of hypnotizability as in *lows* analgesia is not time-locked with suggestions. This finding can be accounted for by a possible strong motivation to analgesia due to the presence of chronic pain, inducing expectation-induced placebo responses following suggestions (Hyland, 2011; Benedetti, 2013; Benedetti and Amanzio, 2013; Carlino et al., 2014) and making them indirectly effective also in *lows*. Thus, the suggestions for analgesia represent an easy and cheap tool for the cognitive control of pain in the large majority of acute (also procedure-related) and chronic pain patients (Elkins et al., 2007; Jensen et al., 2009; Stoelb et al., 2009; Didier et al., 2011; Jensen and Patterson, 2014; Mendoza et al., 2017a,b; Waisblat et al., 2017).

## Interaction of hypnotisability with the behavioral inhibition/activation system and interoception abilities

Recent findings have challenged the established relation between the analgesic effects of suggestions and hypnotizability. In fact, the interaction between hypnotizability and cognitive emotional traits such as those sustained by the Behavioral Inhibition/Activation System (BIS/BAS) (Gray, 1990) in pain imagery (Santarcangelo et al., 2013) and control (Jensen et al., 2016) and in its cortical correlates (Madeo et al., 2015) suggests hypnotizability may be just one of the factors involved in pain control by suggestions of analgesia.

BIS/BAS is based in limbic circuits (Gray, 1990; Angelides et al., 2017), concerns the proneness to approach or withdraw from possibly pleasant and unpleasant conditions, respectively, and is measured by scales (Carver, 2004). BIS is considered an attentional system sensitive to possible punishment, non-reward and novelty, while BAS reflects the motivation to follow one's goals and to approach fun and reward. High BIS is associated with enhanced attention, arousal and vigilance, high BAS with impulsivity, bipolar and attention deficit/ hyperactivity (De Pascalis et al., 2010). In particular, BIS/BAS modulates pain in patients with headache (Jensen et al., 2015) and muskuloskeletal pain (Serrano-Ibáñez et al., 2018).

It has been shown that, even in the absence of significant differences between *highs'* and *lows'* scores, the BISBAS activity masks the hypnotizability-related differences in the vividness of pain imagery (Santarcangelo et al., 2013) and that the activity of BIS/BAS rather than hypnotizability itself is responsible for the hypnotizability-related EEG differences observed during tonic nociceptive stimulation associated and not associated with suggestions for analgesia in *highs*. On the other hand, in chronic pain patients the relation between BIS/BAS and hypnotizability is not linear (Jensen et al., 2016), which indicates a complex interaction.

Another trait potentially influencing the relation between hypnotisability and the effect of the suggestions for analgesia is the ability of interoception that is to detect and interpret bodily states and their changes pre-eminently related to the activity of the autonomic system. Interoceptive signals are monitored and processed at several levels of the central nervous system such as the insula, the orbitofrontal cortex and the cingulate cortex (Critchley and Harrison, 2013) and interoception has been found altered in mental disorders (Murphy et al., 2017; Khalsa et al., 2018) and chronic pain patients (Di Lernia et al., 2016). The role of interoception in pain has been found different in healthy *highs* and *lows*. In fact, a correlation between resting heart rate and pain threshold after suggestions of analgesia has been found in *highs* undergoing cold pressor test, but not in *lows* (Varanini et al., 2018). In addition, preliminary findings indicate higher interoceptive awareness in *highs* than in *mediums* and *lows* (Sebastiani et al., 2018).

Morfo-functional differences between *highs* and *lows* have been observed in the insula and other limbic structures (Landry et al., 2017) and in the cerebellar cortex (Picerni et al., 2018). They consist of reduced gray matter volume (Landry et al., 2017; Picerni et al., 2018) and in a paradoxical increase in pain perception and amplitude of the cortically evoked observed after transcranial anodal stimulation of the cerebellum (Bocci et al., 2017). These morphofunctional differences could sustain the observed hypnotizability-related difference in the role of interoception and of the Behavioral Inhibition/Activation System in pain experience. In fact, interoception contributes to emotion (Critchley and Garfinkel, 2017), the insula and the cerebellum are involved in interoception /interpretation of bodily signals and autonomic monitoring and control, respectively (Di Lernia et al., 2016; Kuehn et al., 2016; Lu et al., 2016; Schulz, 2016; Adamaszek et al., 2017).

## Conclusion

**Figure 1:**
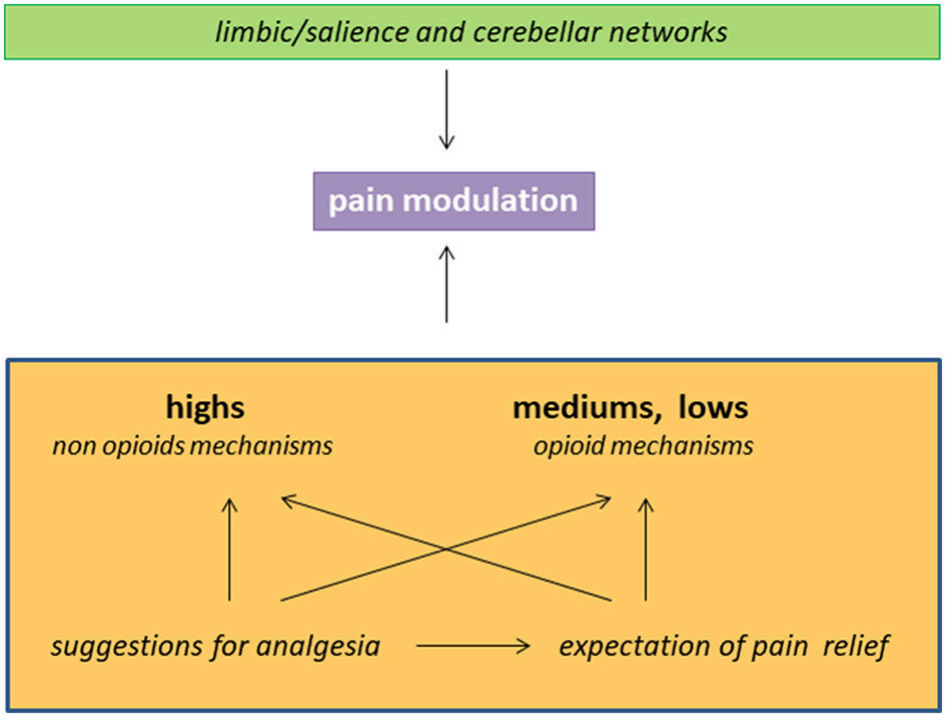
Schematic representation of factors and mechanisms involved in pain reduction in the subjects with different hypnotizability. The interaction between limbic/salience networks and the cerebellum may sustain hypnotisability-related BIS/BAS traits and interoception and influence the response to the suggestions of analgesia. The latter can be associated with expectation of pain relief and act through hypnotisability-related mechanisms.

As summarized in Figure 1, (a) hypnotizability is just one of the individual traits involved in the ability to control pain through suggestions of analgesia; (b) in *highs* any method of cognitive control could be poorly sustained by opioid mechnisms; (c) hypnotizability-related morfo-functional characteristics of limbic circuits and of the cerebellum may sustain differences in cognitive-emotional traits contributing to peculiar pain processing; (d) the efficacy of the suggestions of analgesia in patients with low hypnotizability can be due to placebo responses elicited by suggestions.

The socio-cognitive views of hypnotizability and hypnosis (Lynn and Green, 2011) are the best reference frame to interpret the relation among hypnotizability and pain control. In fact, they allow to consider the joined role of a number of individual traits and of situational variables in pain perception and cognitive control.

## Author contributions

All authors listed have made a substantial, direct and intellectual contribution to the work, and approved it for publication.

### Conflict of interest statement

The authors declare that the research was conducted in the absence of any commercial or financial relationships that could be construed as a potential conflict of interest.
